# Assessment of Bond Integrity, Durability, and Degree of Conversion of a Calcium Fluoride Reinforced Dentin Adhesive

**DOI:** 10.3390/polym13152418

**Published:** 2021-07-23

**Authors:** Mohammad H. AlRefeai, Eman M. AlHamdan, Samar Al-Saleh, Imran Farooq, Eisha Abrar, Fahim Vohra, Tariq Abduljabbar

**Affiliations:** 1 Department of Restorative Dentistry–Operative Division, College of Dentistry, King Saud University, P.O. Box 21069, Riyadh 11475, Saudi Arabia; malrefeai@ksu.edu.sa; 2 Department of Prosthetic Dental Sciences, College of Dentistry, King Saud University, P.O. Box 21069, Riyadh 11475, Saudi Arabia; ealhamdan@ksu.edu.sa (E.M.A.); Salsaleh@ksu.edu.sa (S.A.-S.); fvohra@ksu.edu.sa (F.V.); 3 Faculty of Dentistry, University of Toronto, Toronto, ON M5G 1G6, Canada; imran.farooq@mail.utoronto.ca; 4 Department of Operative Dentistry, Dow University of Health Sciences, Karachi 74200, Pakistan; eshaabrar92@gmail.com

**Keywords:** adhesive, dentin, bonding, calcium, fluoride, spectroscopy

## Abstract

Our study aimed to synthesize and compare the mechanical properties and dentin interaction of two adhesives; experimental adhesive (EA) and EA containing 5 wt.% calcium fluoride (CaF_2_) nano-crystals (CaF_2_ adhesive-CAFA). CaF_2_ nano-crystals were synthesized by reacting two solutions (containing calcium and fluoride) in a glass chamber using a heated air system. The EA was produced using a mix of monomers, photo-initiators, camphorquinone, and electron initiators. The synthesized CaF_2_ nano-crystals were centrifuged to guarantee that inside the adhesive there is homogenized dispersion of the filler particles. Their integration in the EA yielded two groups; Gp-1: EA (without CaF_2_, control) and Gp-2: (5 wt.% CaF_2_ containing adhesive, CAFA). Sixty teeth were prepared and set to form bonded specimens using the two adhesives. The CaF_2_ nano-crystals were irregularly shaped with an average particle size of 30–200 nm. The highest μTBS values were obtained for CAFA-non-thermocycled (NTC) samples (32.63 ± 3.15), followed by EA-NTC (31.80 ± 3.75) specimens. On thermocycling (TC), both adhesive groups presented lower μTBS values (CAFA-TC: 29.47 ± 3.33 and EA-TC: 24.04 ± 3.69). Hybrid layer (HL) formation and resin tags of varying depths were perceived for both adhesive groups. The EDX analysis demonstrated the presence of carbon (C), silica (Si), calcium (Ca), and fluoride (F) for CAFA group. Micro-Raman spectroscopy revealed distinct peaks for CaF_2_ nano-crystals. The CAFA group presented the greatest DC. The addition of CaF_2_ nano-crystals in the adhesive caused improved bond μTBS and DC. The incorporation also demonstrated suitable dentin interaction, depicted by appropriate HL and resin tag development.

## 1. Introduction

Dental caries is a widespread oral disease, with a prevalence of over 50% reported in some countries [1,2]. Carious lesions that cannot be naturally reversed are restored with restorative materials, including glass-ionomer cement (GICs), amalgam, and dental resin composites [3]. Among these materials, resin composites are usually preferred by the patients and dental practitioners due to their distinct advantages, including superior aesthetics, long working time, being mercury-free, and minimally invasive [4,5]. One potential disadvantage of dental composites is their questionable longevity, and a low mean replacement time of 5.7 years [6]. Major factors for the replacement of composite restoration include polymerization shrinkage, marginal leakage, and secondary caries development [7]. Dentin adhesives play a critical role in improving the durability of composite restorations; however, the durability of the adhesion is directly dependent on the polymer’s formed [8]. The clinical success of restoration is also hugely reliant on dentin adhesives’ properties as adhesion forms an intimate bond between the resin and tooth’s hard tissue [9]. Adhesion with dentin is more complex than the enamel because of the wet and more collagenous nature of the former tissue compared with the latter [10]. The adhesive-dentin bond’s quality is hooked with the monomer’s ability to infiltrate the inter-collagen dentinal spaces to establish stable resin tags, consequently developing a steady hybrid layer (HL) [11]. An unstable adhesive-dentin bond faces a loss of strength over time and causes a subsequent failure of the restoration [7]. A strong adhesive-dentin bond is highly desirable as it directly impacts success of the restoration [12,13]. The integration of fillers could improve various adhesive properties, thus improving the longevity of the composite restorations [14]. One such bioactive fillers are calcium fluoride (CaF_2_) nano-crystals.

The role of calcium and fluoride in remineralizing dental tissues is profoundly established [15]. CaF_2_ deposits, which are in contact with the tooth surface, serve as a pool of ions that are leached to promote remineralization, even at low pH [16]. The addition of CaF_2_ nano-crystals makes the material antibacterial, favors fluorapatite formation, and shields the tooth structure against acidic attacks [17,18,19]. Concerning dentistry, the CaF_2_ nano-crystals have been incorporated in different materials, including pit and fissure sealants, GICs, and dentin adhesives and have produced encouraging results [20,21,22]. An earlier study utilized CaF_2_ nano-crystals as fillers inside dentin adhesive and reported remineralization of caries-affected dentin [23]. Mitwalli et al. also observed and reported in their study that CaF_2_ containing nano-composites demonstrated strong antibacterial and ion leaching properties, without any significant effect on the material’s mechanical properties [19].

Considering these beneficial properties of CaF_2_ nano-crystals, we decided to include them in our experimental adhesive (EA) as their integration in the EA could strengthen the adhesive’s mechanical properties. We hypothesized that the addition of these filler particles would boost adhesive’s bond strength, durability, and dentin interaction. Our study had two aims; aim-1: synthesize a novel EA containing CaF_2_ nanoparticles, and aim-2: evaluation of its mechanical properties.

## 2. Materials and Methods

The research ethics review committee of Specialist dental and research center approved the study protocol with No. UDRC-015/2020. All the recommendations of the Helsinki Declaration and its later amendments were strictly followed. Human third molar teeth extracted for orthodontic reasons were collected and inspected under a stereomicroscope (Nikon SMZ800, Tokyo, Japan). Only the teeth, which were free from any apparent defects, were retained after attaining the patients’ written informed consent and were utilized for the experiments in our study. All the teeth were stored in 1% thymol solution and used within one month post-collection.

### 2.1. Formulation of CaF_2_ Nano-Crystals

CaF_2_ nano-crystals were produced first following the earlier recommendations of Xu et al. [24]. Briefly, we synthesized CaF_2_ nano-crystals utilizing a system that used spraying and drying, having a dual liquid nozzle (ViscoMist, Lechler, St. Charles, IL, USA). Two separate solutions; one containing calcium (Ca(OH)_2_ at 2.5 mmol/L) and the other containing fluoride (ammonium (NH_4_) fluoride, at 4 mmol/L) were propelled via the nozzle inside a glass chamber (VM770-48, VM Glass, Vineland, NJ, USA) utilizing a heated air system (at 75 °C) at 10 mL/min feed rate. The two reacting solutions led to the formation of CaF_2_, as explained by the following equation.

Ca(OH)_2_ + NH_4_F → CaF_2_ + NH_4_OH (1)

The above reaction also resulted in the formation of by-product in the form of NH_4_OH, which was then removed as NH_3_ and H_2_O vapors with the air flow.

### 2.2. Formulation of the EA and Incorporation of CaF_2_ Nano-Crystals

The EA was prepared following the previous recommendations of Alqarawi et al. [25]. For its synthesis, a mix of monomers encompassing bisphenol A glycol dimethacrylate (BisGMA), triethylene glycol dimethacrylate (TEGDMA), 2-hydroxyethyl methacrylate (HEMA) and ethyl 4-dimethylamino benzoate and camphorquinone (Esstech Inc., Essington, PA, USA) were used. The composition of our EA involved 50%-Bis-GMA, 25%-TEGDMA, and 25%-HEMA (60%) by weight with ethanol (30% m/m) utilized as a solvent. The photo-initiators that encompassed 0.5% (*n*/*n*) ethyl 4-dimethylamino benzoate and 0.5% camphorquinone were incorporated in line with the monomer moles. Moreover, 1.0% (*n*/*n*) diphenyliodonium hexafluorophosphate (DPIHP) was integrated as an electron initiator to the adhesive mix. This blend was synthesized in a three-necked flask using a magnetic stirrer and a condenser (SA300; Sansyo, Tokyo, Japan). This new EA was secluded in a foil-sheltered dark compartment to avoid photo-polymerization.

The CaF_2_ nano-crystals were added in the EA (5% concentration m/m) to yield an adhesive enclosing 5 wt.% CaF_2_ (CAF adhesive–CAFA). Particles were centrifuged to guarantee that inside the adhesive there is homogenized dispersion of the filler particles. The newly formulated adhesives were left for one day at 37 °C to permit solvent’s evaporation. These adhesives were then secured, placed at 4 °C, and used within three weeks of their formulation.

### 2.3. Characterization of CaF_2_ Nano-Crystals

To characterize the shape of CaF_2_ nano-crystals, we utilized scanning electron microscopy (SEM). A small quantity of CaF_2_ nano-crystals was placed on aluminum stubs and then layered with gold in a sputter-coater (Baltec sputter, Scotia, NY, USA). The micrographs were taken at different magnifications (based on convenience) inside an SEM (FEI Quanta 250, Scanning Electron Microscope, Campanillas, Málaga, Spain) with 10 kV accelerating voltage.

### 2.4. Preparation of Teeth and Procedure for Bonding

Sixty teeth (*n* = 60) were fixed in orthodontic resin (Opti-Cryl, South Carolina, Columbia) at the level of cement-enamel junction within 15 mm (height) segments of polyvinyl pipes (4 mm) and then kept in deionized water. The sample size was based on a previously published similar study [25]. The dentin surfaces of the teeth were exposed with the help of a high-speed handpiece (KaVo Dental Corp., Biberach, Germany) with a diamond disc of 0.15 mm thickness (D943-080, Kerr-Rotary, Duluth, MN, USA). Thirty teeth each (*n* = 30) were randomly allocated to the EA and CAFA group. The teeth surfaces were cleaned using DW in ultrasonic chamber for 5 min. The teeth surfaces were acid etched using phosphoric acid 35% for 60 s following by washing and drying. The adhesives were then smeared onto the exposed dentin surfaces with the help of a micro-brush for 10 s with agitation, which was trailed by air thining for 3 s. This smearing was shadowed by another layer of the adhesive on dentin surface and then photo-polymerization was carried out with the help of a light-curing unit (Eliphar S10; 600 mW/cm^2^ output; 3M ESPE, St. Paul, MN, USA) which was operated from a distance of 10 mm for 20 s. For each tooth, the 2 mm incremental accumulation of resin composite (Filtek Universal; 3M ESPE, St. Paul, MN, USA) was accomplished on the bonded adhesive with the help of a resin mold and metal condenser. This build-up was then cured from all the sides, and excess material was then removed. These bonded teeth were stowed in deionized water for one day at 37 °C. Twenty samples from each group were used for μTBS testing, whereas five samples were investigated for the bond integrity of the resin and dentin interface with the help of SEM and line EDX. The remaining five samples were analyzed for Micro-Raman spectroscopy analysis.

### 2.5. μTBS Testing and Failure Mode Analysis

Twenty bonded samples (ten from each group) were sectioned with a high-speed handpiece (KaVo Dental Corp., Biberach, Germany) with a diamond disc of 0.15 mm thickness (D943-080, Kerr-Rotary, Duluth, MN, USA) to form composite-dentin bonded beams of 1 mm × 1 mm. Seventy beams from each group were produced and these beams were kept in the jaws of a micro-tensile tester (Bisco Inc., Richmond, VA, USA) utilizing cyanoacrylate (Superglue, Louisville, KY, USA). The beams were evaluated while being in tension at a crosshead speed of 0.5 mm/minute until a failure was observed. The failure modes were categorized into adhesive, cohesive, or mixed types and evaluated with the help of a digital microscope (Hirox KH 7700, Tokyo, Japan). Failure was defined as being adhesive when no signs of fractures were detected on the dentin or remnants of resin were seen on the tooth. The failure was classified as being cohesive when complete fracture of dentin or resin was seen and failure of the tooth substrate or failure of the resin composite was observed. The failure was classified as being mixed, when it showed signs of both adhesive or cohesive failure.

Pre-sectioning of the samples, five bonded samples from each group (EA and CAFA) were thermocycled (TC) in water baths at temperatures of 5 and 55 °C for 30 s each with a 5 s dwell time (THE-1100, SD Mechatronik GmbH, Bavaria, Germany) and 10,000 cycles were used. The remaining five samples stayed non-thermocycled (NTC) in both adhesive groups and were kept safe in deionized water for 7 days.

### 2.6. SEM and EDX Mapping of the Adhesive-Dentin Interface

SEM and line EDX mapping was carried out to study the adhesive-dentin interface. Five bonded beam samples from the adhesive groups (EA and CAFA) were polished first using a Polisher (Beuhler Polisher, Lake Bluff, IL, USA) and then washed in an ultrasonic bath (Bandelin Digital- Sigma-Aldrich, Darmstadt, Germany) for 5 min. These beams were further treated with 35% phosphoric acid (Ultra etch Econo Kit- Optident- Yorkshire, South Jordan, UK) for 10 s at the interface and then washed with deionized water for 15 s. The samples were then dipped for 5 min in 5.25%-sodium hypochlorite (NaOCl) solution and cleaned. Treatment with ethanol of concentrations ranging between 80–100% was completed to desiccate the samples. These samples were positioned on aluminum stubs, gold sputter-coated, and analyzed with an SEM (FEI Quanta 250, Scanning Electron Microscope, OR, USA) at 10 kV accelerating voltage.

### 2.7. Micro-Raman Spectroscopy Investigation

Micro-Raman spectroscopy was performed on five remaining samples from each adhesive group. A Micro-Raman spectrophotometer (ProRaman-L Analyzer; TSI, Shoreview, MN, USA) having a related software (Raman reader^®^) was used to acquire Raman spectra(s). The laser beam was protected via a 0.9 objective lens, and 600 mW power and 1 min scan were completed thrice.

### 2.8. FTIR and DC Analysis

FTIR spectroscopy was hired to compute the DC of adhesives (EA and CAFA). These adhesives were evaluated at pre-and post-curing stages. Standardized adhesives were applied on the potassium bromide disc of the spectroscope (Shimadzu, Kyoto, Japan). While the adhesives were in communication with the sensors of the FTIR (Thermo Scientific Nicolet iS20 FTIR spectrometer, MA USA), the absorbance peaks for C-C double bonds were documented for the unpolymerized resin. After polymerizing the adhesive resins for 40 s with a curing light, the FTIR peaks were observed again. With the assistance of an earlier established method [26], C–C aromatic reference peaks (1607 cm^−1^) and C=C (aliphatic) absorbance peaks (1638 cm^−1^) were collected. To determine the DC, FTIR spectra were appreciated between 400–4000 cm^−1^. The adhesive’s transformation rates were calculated using the ratios of C=C and C–C absorbance intensities (% of unreacted double bonds) pre-and post-photo polymerization with the following equation suggested earlier by Al-Hamdan et al. [27].

DC = [1 − (C aliphatic/C aromatic)/(U aliphatic/U aromatic)] × 100%(2)

where, C aliphatic is described as 1638 cm^−1^ absorption peak of cured resin, C aromatic is 1607 cm^−1^ absorption peak of cured resin, Ualiphatic is 1638 cm^−1^ absorption peak of uncured resin and Uaromatic is 1607 cm^−1^ absorption peak of uncured resin.

### 2.9. Statistical Analysis

The findings of μTBS and DC analysis are presented as mean and standard deviation. These values were evaluated statistically utilizing SPSS-20.0 (IBM, Chicago, IL, USA). The normality of the data was first checked via the Kolmogorov-Smirnov test. The ANOVA and post-hoc multiple comparison non-parametric tests were then selected. Statistical significance level was set at 1%.

## 3. Results

### 3.1. Morphology of CaF_2_ Nano-Crystals

**Figure 1:**
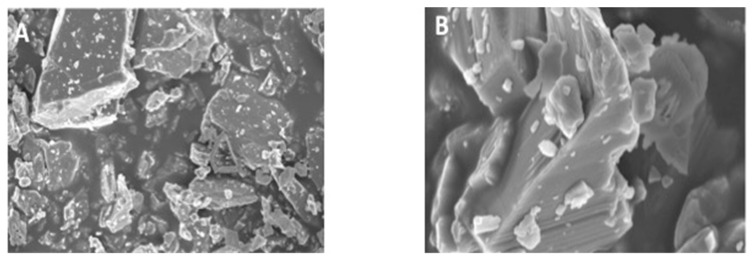
(**A**) Low (8000×) and (**B**) high magnification (50,000×) SEM view of the synthesized CaF_2_ nano-crystals. The nano-crystals exhibited agglomerates of CaF_2_ of various irregular sized polycrystalline grains ranging from 30–200 nm suggesting that they were formed during the process through the fusion of much smaller particles.

The SEM micrographs of CaF_2_ nano-crystals at low and high magnifications are presented in Figure 1A,B, respectively. The CaF_2_ nano-crystals demonstrated agglomeration of non-uniform irregularly sized particles. The average CaF_2_ nanoparticle size ranged between 30–200 nm, proposing that possibly these particles were made due to the fusion of much smaller nano-sized particles.

### 3.2. μTBS Testing and Failure Mode Analysis Outcomes

The highest μTBS values were obtained for CAFA-NTC (32.63 ± 3.15) followed by EA-NTC (28.80 ± 3.75) (Table 1). On TC, both adhesive groups presented lower μTBS values (CAFA-TC: 29.47 ± 3.33 and EA-TC: 24.04 ± 3.69). On intra-group statistical comparison, CAFA-NTC and CAFA-TC values when compared with each other were not significantly different (*p* > 0.01) whereas, EA-NTC and EA-TC values when compared with each other were found to be significantly different (*p* < 0.01). All inter-group statistical comparisons (CAFA-NTC compared with EA-NTC and CAFA-TC matched with EA-TC) were statistically significant (*p* < 0.01) as well.

Concerning failure modes, most of the failures were of the adhesive type (ranging between 80–100% for CAFA-NTC, CAFA-TC, EA-NTC, and EA-TC), followed by mixed-type failures (not exceeding 20% of the total failures) (Table 1). None of the failures observed for any group were of cohesive type.

### 3.3. SEM and EDX Mapping Outcomes

**Figure 2:**
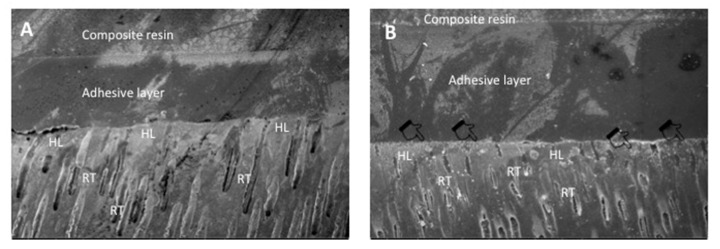
Representative SEM images of the bonded resin dentin interface using (**A**) EA and (**B**) 5 wt.% CaF_2_ modified dentin adhesive (CAFA). Note the addition of CaF_2_ nanocrystals (agglomerations in the image as indicated by pointers) did not significantly affect dentin bonding at the hybrid layer (HL) with well formed resin tags (RT).

**Figure 3:**
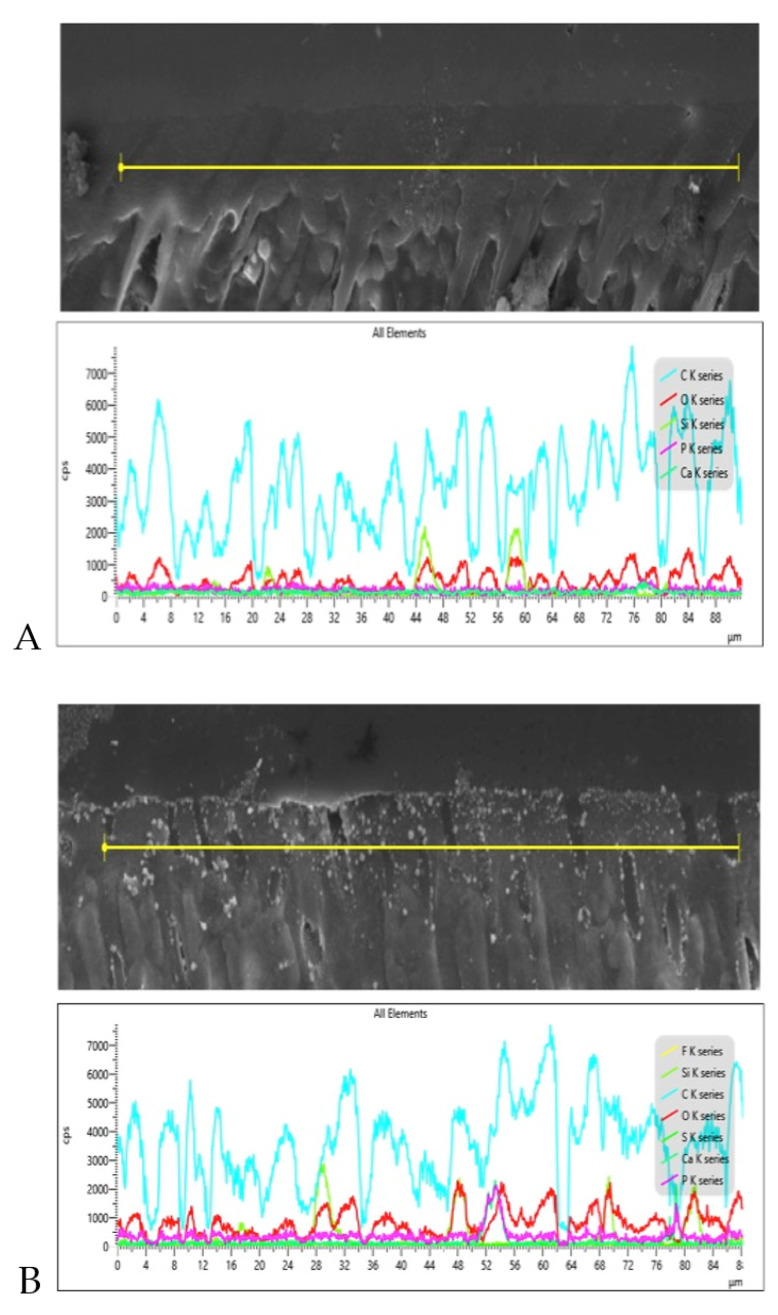
Representative line EDX along the resin dentin interface/hybrid layer for (**A**) EA and (**B**) 5 wt.% CaF_2_ nanocrystal modified dentin bonding agent (CAFA). The modified dentin bonding agent indicates the presence of calcium (Ca), Fluoride (F), and other tooth related elements like silica filler (Si) and carbon (C).

**Figure 4:**
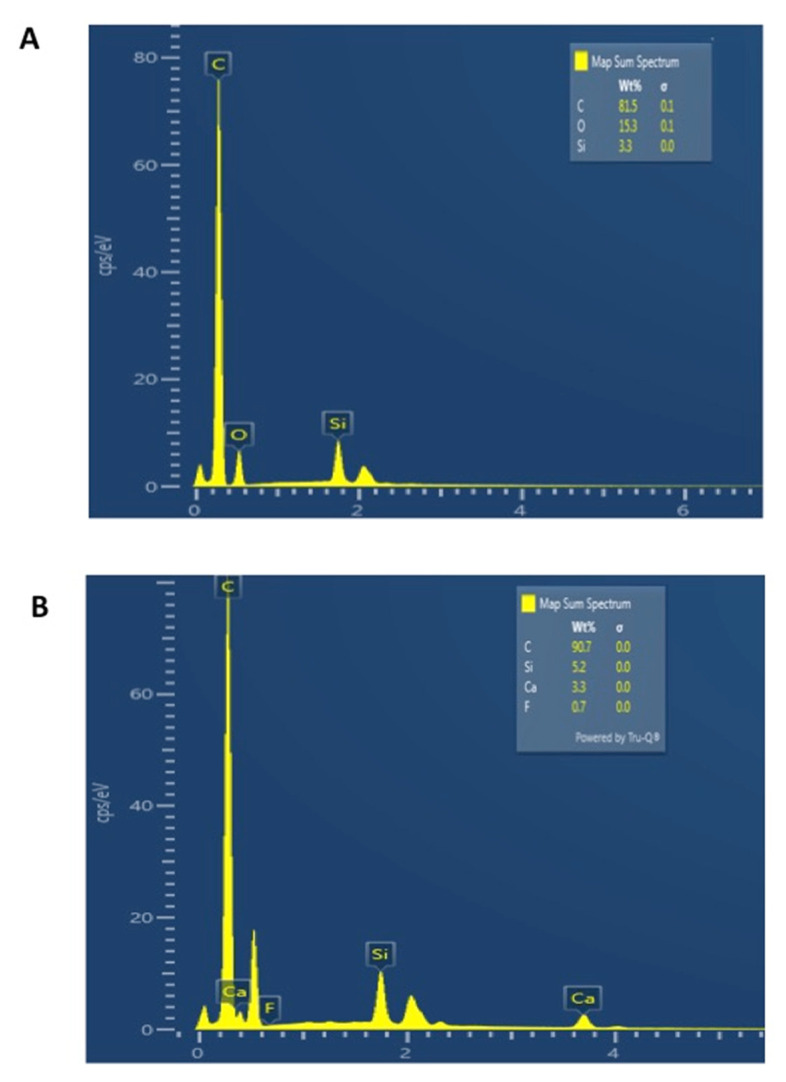
EDX analysis of specific points at (**A**) EA and (**B**) 5 wt.% CaF_2_ nano-crystal modified dentin bonding agent (CAFA). Presence of Silica (Si) Carbon (C) and Oxygen (O) in EA was observed, and EDX graph of CAFA indicates the presence of calcium (Ca), Fluorine (F), Si and carbon (C).

SEM micrographs shown in Figure 2A,B demonstrate bonded resin-dentin interface characteristics for the EA and CAFA groups, respectively. For the EA, HL formation with resin tag development can be observed (Figure 2A). For the CAFA, agglomeration of CaF_2_ nano-crystals was detected at the interface; however, it did not affect dentin bonding at the interface, and resin tags of standard depths were also observed (Figure 2B). The line EDX mapping for the two adhesive groups revealed the presence of essential ions, including carbon (C), oxygen (O) (in the EA group only), and silica (Si) (Figure 3A,B). However, for the CAFA group, certain other ions like calcium (Ca) and fluoride (F) were also witnessed which affirms the presence of CaF_2_ nano-crystals in the adhesive (Figure 4A,B).

### 3.4. Micro-Raman Spectroscopy Analysis Results

**Figure 5:**
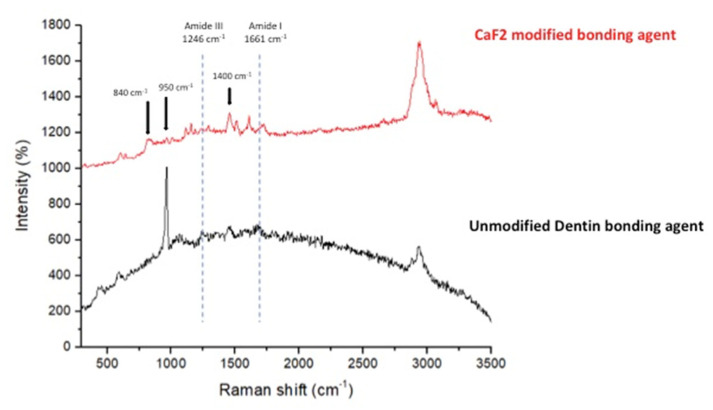
Representative Raman analysis of the EA and CaF_2_ modified dentin bonding agent (CAFA) bonded to tooth structure. The evidence of CaF_2_ existence in the dentin bonding agent was indicated by Raman showing strong bands at 840 cm^−1^ and 1400 cm^−1^ for fluorine and 950 for calcium ions.

The Micro-Raman spectra(s) belonging to CAFA and EA groups were recorded and merged in Figure 5. The CAFA group demonstrated the evidence of CaF_2_ presence in the adhesive, as specified by the existence of strong bands at 840 cm^−1^ and 1400 cm^−1^ for fluorine, and at 950 cm^−1^ for the calcium ions.

### 3.5. FTIR Spectroscopy and DC Analysis Outcomes

**Figure 6:**
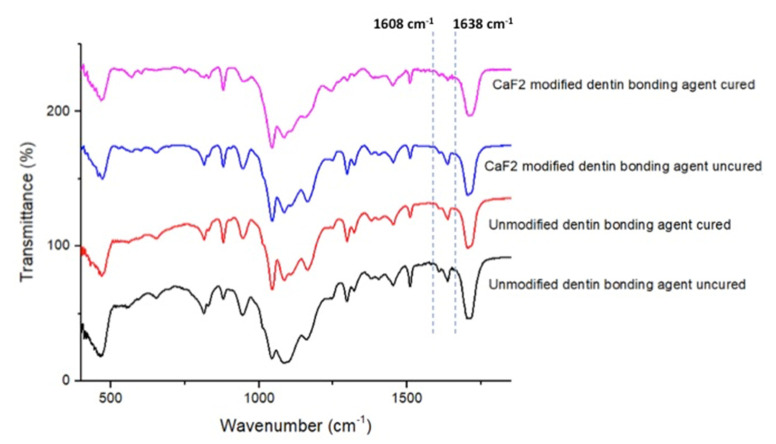
FTIR spectrum of polymerized and unpolymerized EA and CaF_2_ nano-crystal modified adhesive (CAFA). The DC was calculated by estimating the changes in peak height ratio of the absorbance intensities of aliphatic C=C peak at 1638 cm^−1^ and that of an internal standard peak of aromatic C=C at 1608 cm^−1^ during polymerization, in relation to the uncured adhesive as indicated by dotted lines.

The representative FTIR spectra of EA and CAFA groups (cured and uncured) were gathered and combined in Figure 6. The DC was appraised by estimating the disparities in peak height ratio of the absorbance strengths of aliphatic C=C peak at 1638 cm^−1^ and that of a standard inner peak of aromatic C=C at 1608 cm^−1^ while curing, as equated to the uncured adhesive as designated by scattered lines (Figure 6). In terms of DC investigation, the greatest DC was seen for CAFA (61.54 ± 4.07) followed by EA group (56.8 ± 5.5). No statistically significant results (*p* > 0.01) were witnessed upon the comparison of the DC values of the two adhesive groups via Tukey’s test.

## 4. Discussion

The study results indicated enhanced μTBS and DC, and suitable dentin interaction of CaF_2_ containing adhesive. Based on these study findings, the hypothesis that the incorporation of CaF_2_ in the adhesive would augment its bond strength, durability, and dentin interaction was accepted. The addition of bioactive inorganic fillers could augment several mechanical properties of the adhesive, as demonstrated by several previous studies [27,28,29]. Calcium and fluoride are two ions that can remineralize and strengthen the tooth structure [15]. CaF_2_ nano-crystals could serve as a pool of slowly releasing calcium and fluoride ions that could be used to remineralize tooth structure [30]. As secondary caries is one major problem that impacts the longevity of the composite restorations, the incorporation of fluoride-releasing CaF_2_ nano-crystals warrants continued sustained release of fluoride, ensuing an overall anti-caries effect [31]. One main concern with the use of CaF_2_ nano-crystals is their stability; however, they are adequately stable in the oral environment than usually reputed [32]. These beneficial properties encouraged us to incorporate a combination of these two ions in the form of CaF_2_ nano-crystals in our EA and analyze its effect on the adhesive’s properties and dentin interaction.

The CaF_2_ nano-crystals demonstrated irregularly shaped agglomerated particles on the SEM micrographs (Figure 1A,B). Previously, Koeser et al. prepared CaF_2_ nano-crystals for dental applications and demonstrated that they are irregularly shaped when prepared and can present multi-variant morphology (including round, cubic, and hexagonal shapes) [30]. Our morphological findings conform to their study as we also observed irregularly shaped CaF_2_ nano-crystals. However, it would be prudent to mention that it is difficult to predict the exact shape of CaF_2_ nano-crystals after synthesis; therefore, researchers should expect diversely shaped CaF_2_ nano-crystals in their future studies. We also observed HL and resin tag formation for both the adhesive groups (Figure 2A,B). No detectable change was noticed between the two adhesive groups in terms of resin tag formation. The EDX mapping attested to the presence of two essential remineralizing ions (calcium and fluoride) again in the CAFA adhesive (Figure 3). Their presence ensures that the adhesive has remineralizing properties that could be used to augment the strength of the adhesive-dentin bond, ensuring the longevity of the restoration consequently.

The μTBS testing was employed in our study to scrutinize the bond strength of both groups. In an earlier study, it was recommended that when the inorganic fillers are used in the adhesive, their wt.% concentration should not exceed >10%, as it could reduce its bond strength due to a resultant amplified viscosity [33]. In line with this suggestion, we did not add CaF_2_ fillers in our adhesive with a concentration greater than 5 wt.%. Our results demonstrated the greatest μTBS values for CAFA-NTC group. It has been shown before that the materials containing remineralizing ions can release them periodically [34], and this property could have resulted in the improved μTBS for the CAFA seen in our study. Another credible reason explaining this finding could be that materials with nano-remineralizing ions can biomineralize with dentinal collagen fibers [14], causing an augmented remineralization and improved bond integrity, as seen in our study. In our research, adhesive type failures were most commonly perceived. This type of failure generally occurs due to the adhesion loss with fractures not apparent in resin or the dentin [35]. Adhesive type failures are commonly seen in the adhesives containing fillers [27,28], and no single consensual reason has been put forward to explain their occurrence in the literature. To offer a dynamic and aggressive challenge (similar to the oral cavity), we used TC to age the samples and test their μTBS post-aging. According to the ISO standard number 11,405, TC of dental materials within a temperature range of 5 to 55 °C is suitable to offer aging for a limited time [36]. It has been observed in several previous studies that the bond strength decreases after aging [37,38], and our results have echoed similar findings as a decreased μTBS was also observed for the two groups after aging.

The DC analysis of the two adhesives revealed a higher DC for the CAFA group than the EA group. Certain earlier studies have demonstrated that although the integration of inorganic filler particles intensifies the bond strength of the adhesive, it results in a lower DC [28,39]. Our results are in disagreement with their studies as a higher DC for the CAFA group was observed. A higher DC is incredibly desirable as it ensures that an adequate number of monomers are polymerized [40], consequently reducing the chances of nanoleakge and secondary caries development. Compromised DC due to the incorporation of fillers in the adhesive is considered a significant obstacle; however, this finding of our study could positively pave the way for adding inorganic fillers in the adhesives.

The findings of our study are reassuring; still, they should be interpreted with caution. The addition of CaF_2_ nano-crystals in the adhesive led to an escalation in its μTBS and DC and demonstrated suitable dentin interaction. However, a reduced μTBS was observed after the aging. Further studies involving different CaF_2_ filler concentrations should be carried out to establish the impact of various filler concentrations on different mechanical properties of the adhesive.

## 5. Conclusions

The integration of CaF_2_ nano-crystals led to an upsurge in the adhesive’s μTBS and DC. Nevertheless, a decreased μTBS was observed after aging. This filler incorporation also resulted in a suitable dentin interaction, seen in the form of HL and resin tag formation.

## Figures and Tables

**Table 1:** Means and SD for μTBS and failure modes among the study groups.

	μTBS (MPa) (Mean ± SD)			Failure Mode Analysis (%)		
Group (*n* = 10)	NTC	TC	*p*-Value *	Adhesive	Cohesive	Mixed
CAFA	32.63 ± 3.15 ^a A^	-	<0.01	80	0	20
	-	29.47 ± 3.33 ^a A^		100	0	0
EA	28.80 ± 2.75 ^a B^			80	0	20
	-	24.04 ± 3.69 ^b B^		90	0	10

CAFA: Calcium fluoride adhesive, EA: Experimental adhesive, * ANOVA. Dissimilar small alphabets in rows of the same adhesive, denote statistical difference (*p* < 0.01). Dissimilar capital alphabets in the same column denote statistical difference (*p* < 0.01).

## Data Availability

Data of the study is available on request form the corresponding author.
